# Assessment of the Development of Poverty in EU Countries

**DOI:** 10.3390/ijerph19073950

**Published:** 2022-03-26

**Authors:** Agnieszka Sompolska-Rzechuła, Agnieszka Kurdyś-Kujawska

**Affiliations:** 1 Department of Applied Mathematics in Economics, Faculty of Economics, West Pomerania University of Technology Szczecin, Janickiego 31, 71-270 Szczecin, Poland; 2 Department of Finance, Faculty of Economics, Koszalin University of Technology, Kwiatkowskiego 6e, 75-343 Koszalin, Poland; agnieszka.kurdys-kujawska@tu.koszalin.pl

**Keywords:** poverty, sustainable development, outliers, TOPSIS method, EU countries

## Abstract

The aim of the article is to assess of development of poverty in EU countries in 2010 and 2019. The study used the model method of the linear ordering of objects—TOPSIS, considering the distance of each object from both the pattern and the non-pattern development. The originality of the work consists in the use of primary data obtained from the Eurostat database and the winsorized data that were created on the basis of outliers. The indicators characterizing the first goal of sustainable development—“No poverty” were used. The results of the research indicate that the inhabitants of western Europe and Scandinavia are less affected by poverty than the population of eastern and southern Europe. The division of countries according to the scale of poverty is reflected in the level of GDP per capita.

## 1. Introduction

Poverty is one of the greatest challenges in the modern world. It causes misfortune in many people’s lives, limits their fundamental rights, reduces their chances of reaching their full potential, creating high social costs and an impediment to sustainable development [1]. There are countries where poverty affects only a small part of the population, but there are also some where poverty affects a large or even a large part of the population [2]. The United Nations General Assembly more than two decades ago recognized the eradication of poverty as an ethical, political, social, and economic imperative of mankind [3]. The 2030 Agenda, including 17 Sustainable Development Goals (SDGs), adopted in 2015 by 193 states of the United Nations, is an action program of unprecedented scope and importance for the transformation of our world. According to the 2030 Agenda, the current modernization effort of individual countries should focus primarily on eliminating poverty in all its manifestations (SDG1) while implementing a number of economic, social, and environmental goals [4].

Poverty occurs when there is a lack of adequate material resources necessary to cover a certain level of expenditure on goods and services. It leads to people being thrown to the margins, preventing them from using goods and services to the extent that their needs are met [5]. Apart from the lack of income, material goods, and services, the lack of a job, or other sources of income, the phenomenon of poverty also includes the lack of perspectives to change the situation in the foreseeable future [6]. As Abraham and Kumar [6,7] point out, poverty should not be associated only with insufficient income or low consumption. It should be given a broader context because it is also associated with insufficient results in terms of health, nutrition, literacy, handicapped social relationships, insecurity, low self-esteem, and powerlessness. Hence, poverty is a multidimensional phenomenon that occurs both on the economic and social levels. In the first case, it means an uneven distribution of money and material resources, while in the second, insufficient access to goods and services, broadly understood culture, education, health, etc., and small or insignificant participation in public life [5]. The experiences of people living in poverty generally show that they are denied dignity and equality. They live in a closed circle of powerlessness, stigmatization, discrimination, exclusion, and material poverty, all of which reinforce each other [8].

There can be many causes of poverty. The explanation of the phenomenon of poverty is based on three theories: behavioral, structural, and political [9]. Behavioral theories focus on individual behavior driven by stimuli and culture [10]. According to these theories, the poor are poor because they engage in unproductive, poverty-increasing behaviors or threats [11]. The source of poverty is, inter alia, a low level of education caused by the reluctance to learn and acquire knowledge, obsolescence, and inability of a person to ensure old age, self-replication of poverty due to the reluctance or inability to exit the vicious cycle of poverty, low level or lack of social and family ties, which can provide material support, asocial qualities and behavior of a given person, etc. [12]. Structural theories relate to the macro and meso demographic level and the economic context representing the available opportunities and limitations [13]. According to these theories, poverty is about a dysfunctional economic, political and social system that causes people to have limited opportunities and resources [14]. The causes of poverty include unfavorable economic conditions, counting economic crises, political instability and various conflicts, ecological disasters and climate deterioration, a decline in income levels, and the existence of regions and sectors of the economy with traditionally low income. Moreover, the causes of poverty include the inability to obtain a good education or access to health care, which results in the inability to fully engage in activities aimed at generating income [12]. Political theories, on the other hand, show that governments and institutions create policies that cause poverty and alleviate the relationship between behavior and poverty [10]. The source of poverty is a strong dependence on decisions made by various officials and the bureaucratic mechanism created by them, improper policy of regulating economic activity, lack or incorrect operation of the mechanism of redistribution of income, etc. [12]. The theory of exchange may be an interesting supplement to contemporary theories of the causes of poverty. Poverty is the result of a disturbance in social exchange, which is a permanent feature of social life [15,16]. At the root of the causes of poverty, according to Schiller [16], are: flawed character, restricted opportunity, and Big Brother. Flawed Character refers to moral flaws or the character of an individual, such as promiscuity and laziness that cause poverty. The basis of the defective character is the individualistic approach to poverty in which poverty is under the control of the individual. Restricted opportunity focuses on external factors that are beyond the control of the individual [16]. Poverty follows an economy where a lag in the economy results in fewer jobs and more poverty. Discrimination deprives certain groups of the possibility of gaining the human capital necessary to achieve economic progress [17]. Big Brother refers to the role of government in creating incentives to remain poor. The government destroys incentives for stable families and economic self-sufficiency through high taxes or social benefits [16].

Combating poverty and social exclusion has become one of the most important social goals of the European Union and its member states. In the Europe 2020 strategy for jobs and smart, sustainable, and inclusive growth, EU leaders committed to taking action to reduce the number of Europeans living below the national poverty line by 25% and lift more than 20 million people out of poverty by 2020 [18]. Despite the general wealth of the European Union, more than 20% of the population in Europe is currently at risk of poverty or social exclusion. About 1 in 6 live in a household with disposable income below the national poverty line. A total of 1 in 17 people is severely materially deprived, meaning that their living conditions are severely affected by a lack of resources, such as not being able to pay their bills, keeping their home warm, or taking a week’s vacation away from home. About 1 in 12 people live in households with very low work intensity. The richest 20% of households in Europe earn five times more than the poorest 20% of households [19]. This situation may delay the achievement of Europe’s Sustainable Development Goals. Focusing on eradicating poverty in all its forms is the most promising strategy that could ultimately usher in a positive cycle of progress toward the SDGs [20]. According to the studies of Pradhan et al. [21], poverty eradication (SDG1) has a synergistic relationship with most of the other SDGs. Poverty reduction is linked to advances in SDG 3 (good health and good self-feeling), SDG 4 (high-quality education), SDG 5 (gender equality), SDG 6 (clean water and sanitation), SDG 10, among others, (reduce inequalities), SDG 12 (responsible consumption and production), and SDG 13 (climate action). This forces the government to intensify efforts to combat poverty more effectively. For example, a family that no longer suffers from extreme poverty (SDG1) will be able to lead healthier lives for themselves and others by stopping the spread of infectious diseases (SDG 3), contributing to a stronger economy (SDG 8) increasing delivery modalities through tax levies (SDG 17), which in turn will enable public investment in infrastructure (SDG 9) that will provide education and other important services (SDG 4) [20].

An appropriate information system is a necessary condition for the programming and implementation of an effective policy of combating poverty, both in the economic and social sphere. Hence, it is advisable to constantly analyze this phenomenon in order to diagnose and compare the situation in individual countries as precisely as possible. Thanks to this, it will be possible to monitor the progress in eradicating poverty by individual governments of EU countries.

Determining the level and scale of poverty in order to eliminate its causes and counteracting its negative effects is a significant challenge for social policy in every country. Moreover, it is extremely important to monitor this phenomenon, which is emphasized by many international institutions. As a consequence, it resulted in the launch of many initiatives related to the analysis of this phenomenon, its measurement, and methods limiting its negative effects [22]. The greatest controversies in measuring poverty at the national or regional level are related to the way of determining the level of satisfaction of needs deemed desirable, i.e., the way poverty is understood [23]. Poverty can be understood in an absolute or relative way. In absolute terms, poverty is based on the notion of the degree of satisfaction of needs, defined in specific quantitative and valuable categories [24]. It consists in estimating the “market basket” of goods and determining an absolute poverty line that is the cost of purchasing these goods for households of various sizes [25]. The category of poverty in relative terms is based on the ratio of the level of meeting the needs of individuals (people, families, households) to the level of meeting these needs by other members of society [24]. The poverty line is established on the basis of the income distributions of the reference population, the national population in general, and a point in that distribution is established below which individuals are considered poor. The main interpretation issue with relative poverty lines is that poverty rates based on them may remain constant or even fall if all households (including the poorest ones) experience a decline in their incomes. Moreover, people identified as poor in one country might not be considered poor in another, given the substantial differences in median incomes across OECD countries [26]. An equally important element in the measurement of poverty is the definition of the poverty criterion. Since the late 1990s, the OECD has produced indicators on income inequality and poverty based on a set of common definitions, classifications, and data treatments. The indicators are based on the most appropriate data source available in each country. All the indicators collected by the OECD are based on the concept of “equivalised” household disposable income. Relative poverty is measured by size indices (i.e., percentage of the population with incomes below the poverty line) based on different median income thresholds for both the national population (national poverty lines) and the regional population (regional poverty lines) [27]. In turn, the European Commission uses three partial indicators to monitor the achievement of the goal of reducing the number of people at risk of poverty or social exclusion. The first one reflects the risk of relative poverty and the indicators concerning people at risk of severe material deprivation and people living in households with very low work intensity. These indicators are determined on the basis of data from EU Statistics on Income and Living Conditions—EU-SILC [26]. As noted by Panek and Zwierzchowski [28], the inclusion of non-material indicators in the poverty assessment is a significant step toward a more complete assessment of poverty in EU countries. The measurement of material deprivation, as opposed to the measurement of monetary poverty, is carried out according to the absolute approach, in which the assessment of the financial situation of households is not based on the relation of their level of deprivation to the level of deprivation of other households. This approach ensures comparability of the living situation between EU countries. The Employment, Social Policy, Health and Consumer Affairs Council (EPSCO) indicates that a poor person is one who is characterized by at least one of the two criteria of poverty: financially poor or subject to material deprivation (non-monetary poor). This indicates the need to consider in the assessment of the financial possibilities of satisfying one’s needs by the audited entity (person, household) not only its financial resources in the form of current income but also income from previous periods and in the form of accumulated resources [29]. The method of identifying the poor recommended by EPSCO may be difficult to apply in the EU countries. This is due to the fact that EPSCO does not treat the EU as a single body but assesses poverty in individual EU countries independently. This leads to an overestimation of the scope of poverty in affluent countries (with high-income equivalents) and its underestimation in the least prosperous countries [24].

Among the currently used methods of statistical poverty measurement, two basic approaches can be distinguished: classical and multidimensional. The first of them, treating poverty as a lack of sufficient economic resources to meet the basic needs of an individual (mainly in the existential sense), is characterized by adopting a single measure in the study of poverty (most often a specific level of household income or its necessary expenses). In the multidimensional approach, in order to identify the poor population, in addition to the basic determinants of the economic situation of households (appropriate to the classical approach), non-monetary indicators are also used, such as: equipping households with durable goods, forms of spending free time or even indicators for assessing the state of health [30].

The aim of this article is to assess the development of poverty in EU countries in 2010 and 2019.

This article is a contribution to research on the assessment of the development of poverty, using the method of multivariate comparative analysis, linear ordering of TOPSIS objects on the basis of winsorized data.

The multifaceted nature of poverty forces the need to apply multidimensional approaches to measuring this phenomenon. The traditional one-dimensional approach measures poverty using the monetary indicator, while the multidimensional approach sees the experience of poverty in poor people as many attributes that deprive a person of the fulfillment of their needs [31] provides an effective and systematic diagnostic tool.

## 2. Data and Methods

### 2.1. Data

The poverty level survey was carried out in relation to EU countries, based on Eurostat data for 2010 and 2019 [19]. In the assessment of poverty, the indicators available in the Eurostat database were used as the first goal of sustainable development (no poverty). Table 1 presents the indicators and their definitions given in the Eurostat database [19].

Later in the article, indicators 1–10 were subjected to statistical analysis. The basic descriptive parameters were calculated, and then comparative analysis of the poverty level in EU countries in 2010 and 2019 was carried out. Linear ordering of countries was created on the basis of indicators 2–10. Indicator 1 is not included in the ordering of countries because indicators 2, 3, and 4 are its component parts.

### 2.2. Methods

One of the methods of linear object ordering—TOPSIS (Technique for Order Preference by Similarity to an Ideal Solution) was used to achieve the goal. The classical TOPSIS method was first presented by Hwang and Yoon [32]. The TOPSIS method is the reference method and consists in calculating the Euclidean distances of each assessed object from both the pattern and non-pattern development.

The TOPSIS method consists of the following steps [33,34,35]:

1. Selection of variables on the complex phenomenon;
2. Determination of the impact direction of variables in relation to the complex phenomenon;
3. Normalization of the variable values;
4. Determine the positive ideal (PIS) and negative ideal (NIS) solutions;
5. Calculating the distance of all alternatives to the PIS (*A*^+^) and the negative ideal (*A*^−^) solution, using the Euclidean distance;
6. Determination of the value of a synthetic measure;
7. Linear ordering of object and identification of developmental types.

The selection of features is made on the basis of content-related and statistical analysis [36]. The statistical analysis considers the variability of the objects in terms of each variable (it should be large) and the degree of correlation between the variables. In the second stage, the type of the variable is determined, which may be stimulating, destimulating, or neutral in relation to the examined criterion (e.g., the level of poverty). The next stage of building a synthetic measure is the normalization of the features. It leads to the deprivation of the measurement results and the unification of the orders of magnitude of the features. Among the many methods of normalization, the method of zero unitarization deserves attention because this technique provides normalized values by linear transformation and keeps relationships between original data [37,38]. The normalization of the variables is performed according to the following formulas [37,38]:

- For stimulant:

(1) $$z_{ij}=\frac{x_{ij}-\underset{l}{\min}x_{lj}}{\underset{l}{\max}x_{lj}-\underset{l}{\min}x_{lj}}\;\left(\underset{l}{\max}x_{lj}\neq \underset{l}{\min}x_{lj}\right),$$

- For destimulant:
  (2) $$z_{ij}=\frac{\underset{l}{\max}x_{lj}-x_{ij}}{\underset{l}{\max}x_{lj}-\underset{l}{\min}x_{lj}}\;\left(\underset{l}{\max}x_{lj}\neq \underset{l}{\min}x_{lj}\right),$$
  where: $z_{ij}$—standardized value of the *j*-th feature (j=1, 2, …, k) for the *i*-th object (i=1, 2, …, n), *n*—number of object.

In the zero unitarization method, a constant reference point is assumed, the range of the normalized variable. The use of this method makes the range of the normalized feature constant and amounts to one. The normalized feature assumes values in the range [0,1]. Moreover, this method makes it possible to normalize the features taking positive, negative, and zero values [39]. This technique provides normalized values by linear transformation and keeps relationships between original data [40].

In the fourth stage, the coordinates of the model units, the pattern and the non-pattern of development, are established. The values of the pattern (*A^+^*) and the non-development pattern (*A^−^*) are defined as:

(3) $$A^{+}=\left(\underset{i}{\max}\left(z_{i1}\right),\;\underset{i}{\max}\left(z_{i2}\right),\;\ldots ,\;\underset{i}{\max}\left(z_{ik}\right)\right)=\left(z_{1}^{+},z_{2}^{+},\;\ldots ,z_{k}^{+}\right)$$

(4) $$A^{-}=\left(\underset{i}{\min}\left(z_{i1}\right),\underset{i}{\min}\left(z_{i2}\right),\;\ldots ,\;\underset{i}{\min}\left(z_{ik}\right)\;\right)=\left(z_{1}^{-},z_{2}^{-},\;\ldots ,z_{k}^{-}\right)$$

If zero unitarization is used as the normative formula, it is:

(5) $$z^{+}=\underset{k}{\underset{⏟}{\left(1,\;1,\;\ldots ,\;1\right)}}z^{-}=\underset{k}{\underset{⏟}{\left(0,\;0,\;\ldots ,\;0\right)}}$$

In the next stage, the Euclidean distances of each object from the pattern and non-pattern are calculated according to the following formulas:

(6) $$d_{i}^{+}=\sqrt{\overset{k}{\underset{j=1}{\sum }}{\left(z_{ij}-z_{j}^{+}\right)}^{2}}$$

(7) $$d_{i}^{-}=\sqrt{\overset{k}{\underset{j=1}{\sum }}{\left(z_{ij}-z_{j}^{-}\right)}^{2}}$$

On the other hand, the value of the synthetic measure is determined as follows [23]:

(8) $$\mu_{i}=\frac{d_{i}^{-}}{d_{i}^{+}+d_{i}^{-}}$$

where:$\;0\leq \mu_{i}\leq 1,\;i=1,\;2,\;\ldots ,\;n$.

The smaller the distance of a given object from the development pattern and thus greater than the development non-pattern, the closer the value of the synthetic measure. After ordering the value of the aggregate feature, four classes are determined based on the arithmetic mean and the standard deviation calculated from its values:

- Class I: $\mu_{i}\geq \bar{\mu }+s_{\mu }$—very high level
- Class II: $\bar{\mu }\leq \mu_{i}\leq \bar{\mu }+s_{\mu }$—high level
- Class III: $\bar{\mu }-s_{\mu }\leq \mu_{i}\leq \bar{\mu }$—medium level
- Class IV: $\mu_{i}<\bar{\mu }-s_{\mu }$—low level
  where: $\bar{\;\mu }$—arithmetic mean, *S^μ^*—standard deviation of the value of the synthetic measure.

The correct interpretation of the results of the conducted analyzes depends to a large extent not only on the completeness and absence of errors in measuring statistical data but also on the presence of atypical objects in the observation set. These objects are also called outliers and are often also referred to as noise, error, exception, singularity, or imperfection. Outliers have a large impact on the results of the analysis. The occurrence of remote observations is not always a negative phenomenon. Distant observations may be the result of measurement errors and the result of correct measurements and illustrate the true, though rare and unusual behavior of the phenomenon under study. In the latter case, these observations should definitely not be removed, as their information content is usually very high. In both cases, it is important to identify outlier observations and treat them appropriately.

The issue of outliers is raised by many researchers who present different ways of dealing with outliers [40].

Aguinis et al. [41] provide evidence that different ways of defining, identifying, and dealing with outliers change substantive research conclusions. As the literature review shows, researchers define outliers in different ways and use different methods in managing them. As Hawkins [42] reports: “The intuitive definition of an outlier would be “an observation which deviates so much from other observations as to arouse suspicions that it was generated by a different mechanism”. As we know, outliers also have a large impact on the values of descriptive parameters, in particular classical parameters, such as: arithmetic mean or variance. If the value of the outlier variable results from a measurement error, it should be considered a negative phenomenon. However, in many situations, distant observations are the result of correct measurements and reflect the true, though rare and atypical behavior of the studied phenomenon [43]. In such a case, these observations should not be deleted, as generally, their informative content is very high. The best solution is to identify the cause of the outliers and take steps to improve the quality of the data. A box plot is a very helpful tool in the identification of outliers, which very well indicates the existence of such values and is based on the quantile criterion. In such a situation, the value of a single variable is considered remote if it is outside the range [33]:

(9) $$\langle Q_{1}-1.5\cdot Q;\;Q_{3}+1.5\cdot Q\rangle$$

where: $Q_{1},\;Q_{3}$—the first and the third quartiles of the *j*-th variable, Q—are the interquartile range of the *j*-th variable.

Another way to proceed, using the method of the linear ordering of objects, in the case of the occurrence of distant values and thus the occurrence of asymmetry of distribution is to use the Weber median as a vector that minimizes the sum of Euclidean distances from given points representing the considered objects. Thus, it is somewhat “in the middle” of them but at the same time resistant to the occurrence of outliers [39,44,45]. Winsorised data is one of the methods of dealing with the problem of outliers in the distributions of variable values. The variables describing the level of poverty in individual EU countries are characterized by asymmetry, sometimes quite strong, and the presence of outliers. These observations can influence the results of the linear ordering of countries. In the case of using the TOPSIS method, when the maximum and minimum values of the variables correspond to the values of the pattern (*A*^+^) and the non-pattern of development (*A*^−^), it is possible to get these values away from the typical ones, which results in narrowing the range of variability of the synthetic measure, which in turn makes it difficult to correctly identify objects in terms of the adopted criterion [33]. An important issue is to pay attention to whether there are outliers in the set of variables and to improve the quality of these values. In this study, after identifying outliers, a one-dimensional approach based on the quartile criterion was used to improve them.

## 3. Results

The poverty assessment was carried out for the 27 EU countries in 2010 and 2019. 

**Figure 1:**
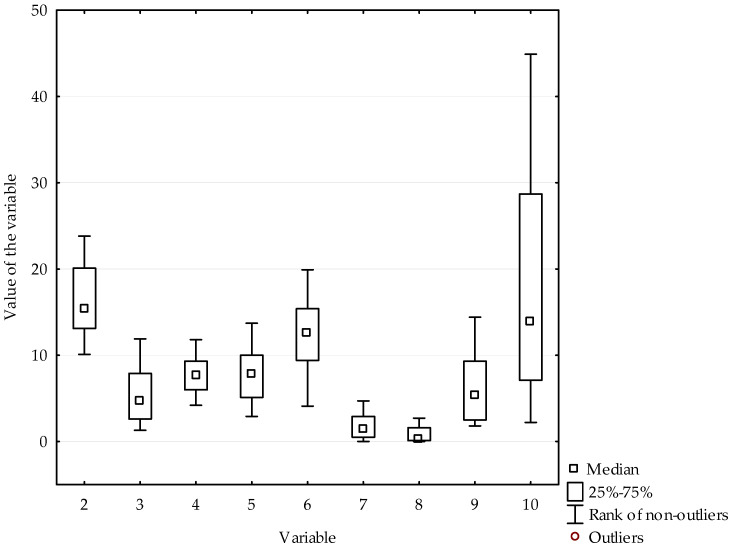
Distributions of variables with winsorized data.

The variables used in the assessment of poverty are characterized by strong asymmetry and the occurrence of outliers, e.g. variable distribution population having neither a bath, nor a shower, nor indoor flushing toilet in their household by poverty status is characterized by very strong asymmetry and outliers have been observed in the case of countries such as Bulgaria, Greece or Lithuania. The use of the method of linear ordering of countries with outliers may contribute to incorrect identification of poverty levels. Therefore, the impact of asymmetry in variable distribution should be limited by using methods that allow replacing outliers with constant values. This process is called winsorisation. The results obtained on the basis of winsorised data are presented later in the article. The distributions of the variables, after replacing the outliers with constant values, were characterized by weak or moderate asymmetry and negative kurtosis, which proves a lower intensity of extreme values. Figure 1 shows the distributions of variables, which are the basis of the linear ordering of countries after replacing the outliers. 

On the basis of the information in Table 2, it can be concluded that the average values of the indicators in 2019 decreased compared to the values in 2010. The largest percentage of the population in EU countries lives in an overcrowded household and experiences housing deficits.

In 2019, we continued to see a high proportion of people in EU countries at risk of poverty. The analyses show that Romania is a country with four most unfavorable values in 2019, referring to: people at risk of poverty or social exclusion, in work at-risk-of-poverty rate and population having neither a bath, nor a shower, nor indoor flushing toilet in their household by poverty status and overcrowding rate by poverty status. Another country with the two highest values for: severely materially deprived people and population unable to keep home adequately warm by poverty status is Bulgaria. In Estonia, the self-reported unmet need for medical examination and care by sex indicator was over six times higher than the average for all countries (15.5%). On the other hand, for Luxemburg, the lowest value of the indicator severely materially deprived people was recorded. In the case of Malta, the following indicators: self-reported unmet need for medical examination and care by sex and population having neither a bath, nor a shower, nor indoor flushing toilet in their household by poverty status was at the level zero.

In the next step, the values of the variables were normalized by means of zero unitarization. As a result of this transformation, the variables take the values from the numerical interval 0;1. Based on the normalized values of the variables for winsorized data, the positive and negative ideal solutions distance for each country was calculated. Then, the values of the synthetic poverty measure were calculated using the TOPSIS method, and four types of classes of countries with a similar poverty level were identified (Table 3).

The lowest poverty level in 2010 was recorded in countries such as Czechia, Denmark, Finland, Netherlands, and Sweden. These are the countries where the variables have the lowest values among the studied countries, e.g., Czechia has the lowest percentage of people at risk of income poverty after social transfers (9.0%), and Netherlands and Sweden have the lowest percentage of the population having neither a bath, nor a shower, nor indoor flushing toilet in their household by poverty status (0.0%). On the other hand, in the countries of Bulgaria, Latvia, Lithuania, Poland, and Romania, a very high level of poverty was observed. In Romania, the worst-case value for three variables was recorded in 2010: people at risk of income poverty after social transfers (21.6%), in work at-risk-of-poverty rate (17.9%), and population having neither a bath, nor a shower, nor indoor flushing toilet in their household by poverty status (38.5%). Low poverty levels in 2019 were recorded in: Austria, Czechia, Germany, and Malta. A very high level of poverty was observed in: Bulgaria, Greece, Latvia, Lithuania, and Romania. The level of poverty, in 2019, in Bulgaria, Latvia, Lithuania, and Romania did not improve and was the highest among EU countries. In the case of Greece, poverty levels deteriorated to very high in 2019. In the period from 2010 to 2019, a significant increase in the value of indicators such as: severely materially deprived people (from 11.6% to 16.2%), people living in households with very low work intensity (from 7.6% to 13.8%), and self-reported unmet need for medical examination and care (from 5.5% to 8.1%). In several countries, there has been an improvement in poverty levels, e.g., in Denmark, Finland, Germany, Malta, and Poland.

The positions of countries in the rankings obtained on the basis of indicators are significantly (p<0.5) and strongly related (the value of the Kendall rank correlation coefficient is 0.721).

In 2010 and 2019, the percentage of countries with very high or high levels of poverty was the same at 48,1%. In 2019, the percentage of countries with low levels of poverty decreased to 14.8%.

Table 4 shows the typological classification of EU countries in terms of poverty levels in 2010 and 2019.

**Figure 2:**
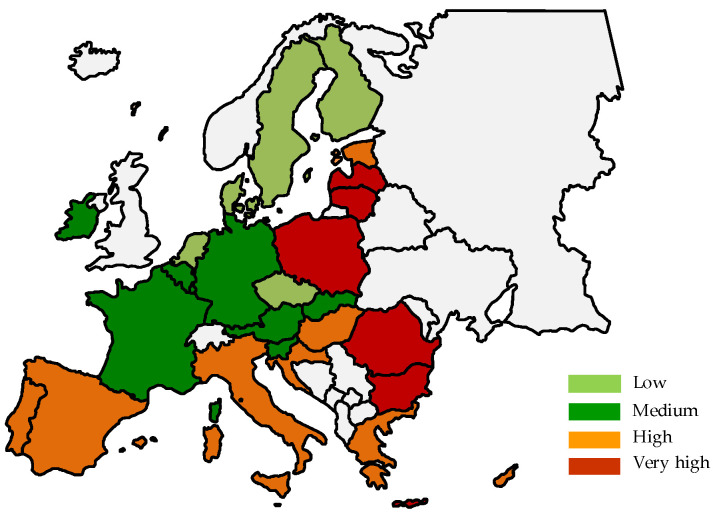
Spatial delimitation of types of EU countries in terms of poverty level in 2010.

**Figure 3:**
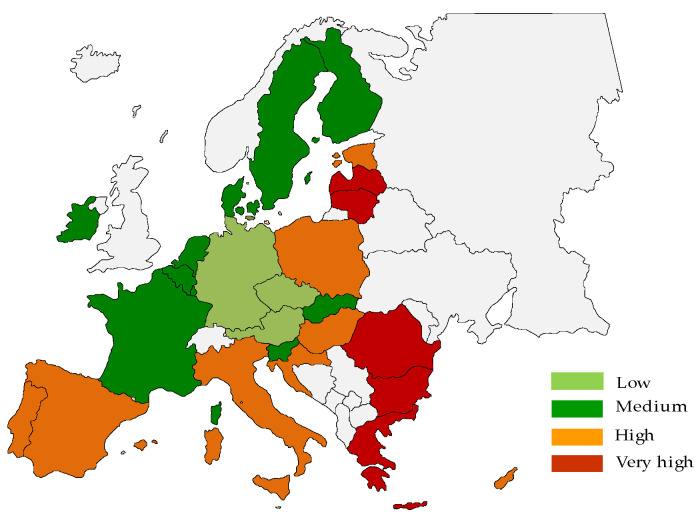
Spatial delimitation of types of EU countries in terms of poverty level in 2019.

The spatial delimitation of countries indicates that countries with low or medium poverty levels are located mainly in western Europe and Scandinavia (Figure 2 and Figure 3). On the other hand, the group of countries with a high or very high level of poverty is the countries of eastern and southern Europe.

Comparing the level of poverty to the value of GDP per capita in EU countries, it can be stated that in the group of countries with a very high level of poverty, the lowest level of GDP per capita in PPS as a percentage EU average of 81.33% was recorded. In countries with high poverty levels, the percentage of GDP per capita in PPS was slightly higher, 83.89%. Much higher values of GDP per capita in PPS as a percentage EU average were recorded in countries with a low or very low level of poverty, amounting to 140.00% and 125.00%, respectively.

## 4. Discussion

Poverty is a complex and pressing problem that has been gaining attention all over the world for a very long time. Researchers focus on several overlapping dimensions of poverty: uncertain livelihoods, excluded places, physical problems, gender relations, problems in social relations, insecurity, abuses by the rulers, incapacitation of institutions, weak social organizations, and limitations of the poor [46]. It is, therefore, no surprise that eradicating poverty in all its forms has become the first and foremost goal of sustainable development. Moreover, it has been proven that its implementation determines the effectiveness of the implementation of most of the remaining 16 Sustainable Development Goals [20,21]. In recent years, a number of different sources of poverty have been proven, which should be seen in the individual “contaminated” features of the human character, the restricted opportunity, and resources of the economic, political, and social system, as well as the influence of the government, which in the role of Big Brother creates incentives to remain poor [16]. This makes eradicating poverty a completely difficult endeavor. Policymakers in European countries have recently taken a number of initiatives to lift more than 20 million people out of poverty by 2020 [18]. It is already known today that this goal has not been achieved, and EU leaders reformulated it in March 2021. The Roadmap to the European Pillar of Social Rights has a new target of reducing the number of people living in poverty by at least 15 million by 2030 [18].

This study provides evidence of different levels of poverty in selected EU countries. When analyzing all the discussed indicators together, it can be noticed that the situation in the area of poverty reduction in the EU countries has been gradually improving in recent years, but it is still not satisfactory in some countries, or it has worsened. Only a few EU countries have managed to achieve a low level of poverty, and what is more, only a few countries have significantly improved in the last decade.

Considering the situation in individual countries, it is worth noting that in six of them, Bulgaria, Romania, Lithuania, and Latvia, the countries of Eastern Europe, and in Greece and Italy, the countries of southern Europe, the highest levels of poverty were recorded. In Bulgaria and Romania, almost every third person is at risk of poverty or social exclusion, as is in Latvia and Lithuania. At the same time, in these countries, income inequality, measured by comparing the income of 20% of the richest households with the income of the poorest 20% of households, is the highest in the entire EU (the highest rate was recorded in Bulgaria, which means that in 2018 the richest 20% households had an income almost eight times higher than the 20% of the poorest households in the country; the EU average is 5.1). In these countries, the reasons for the high risk-of-poverty rate lie in the weakness of the redistribution of funds within the tax and social security systems, which reduce income inequalities less effectively than in other EU countries. In addition, since the crisis of 2008, these countries have weakened the capacity of tax systems to counteract growing market inequality [47]. In Italy and Greece, poverty levels have worsened significantly. This indicates that eradicating poverty in these countries remains a major challenge. The reasons for the unsatisfactory improvement in the poverty area in these countries are seen in particular in the financial crisis of 2008. Italy and Greece are among the most affected by the financial crisis. Moreover, in these countries, the economic recovery after the crisis was the slowest compared to other European countries [48,49,50].

The financial crisis and its effects have led some governments to cut government welfare spending. Deteriorating macroeconomic conditions and negative fluctuations in output meant that euro area countries had limited capacity to intervene to lift people out of poverty due to lack of resources. In fact, it has occurred that some countries, after using fiscal policy as a stabilization instrument in the aftermath of the crisis, were forced to implement fiscal austerity measures [51]. The countries worst affected by the crisis faced sharply declining private and public incomes and declining growth rates due to falling foreign capital inflows, domestic credit and remittances, falling commodity prices, and deteriorating trade conditions. Mobilizing additional resources, and even maintaining existing levels in such a context, has proved to be a major challenge [52]. As a result, the dynamics of poverty changed. Very poor groups of the population may not initially experience a significant deterioration in their situation because their situation was already tragic, and many were already detached from the labor market. With unemployment rising and wage arrears growing, new sections of the population have become prone to poverty. The poor coverage of unemployment benefits on the one hand and the very narrow targeting of government social welfare on the other left many of the newly poor without any support to cover the loss of income [53]. In Greece, the consequences of the global financial recession and the applied austerity measures created a difficult situation, the burden of which was not only visible in the economic sphere but also had a significant negative impact on the national health sector and social services [54]. The government, on the other hand, was not able to support the public health service sufficiently and maintain the already declining social services [55]. These events have their long-term consequences, which are reflected in the still very high level of poverty. According to OECD data, Greece still maintains a large wealth gap between the richest and poorest people in Europe [56]. Moreover, there is evidence that poverty in Greece is to some extent “self-perpetuating”, that is, when people fell below the poverty line, they tended to remain in poverty longer, regardless of their characteristics [57,58].

The very high level of poverty in some EU countries since 2010 seems to run counter to the community’s ambitious goals in reducing poverty. A consequence of such a state of affairs may be the level of government expenditure allocated to combating poverty. It should be noted that it is not only the amount of these expenses that are important but the degree and areas of their targeting and the method of financing. For example, in Finland, Ireland and Denmark, as a result of social transfers, the number of people at risk of poverty decreased by more than half in 2017. This means that social transfers in these countries reached the most vulnerable people, thus effectively implementing the first goal of sustainable development. Whereas in Greece and Romania, the reduction was below 20%, despite the fact that in these countries, much more funds are spent on social protection (as a percentage of GDP) [59].

In other Eastern European countries (Estonia, Poland, Slovakia, Hungary), a high level of poverty was recorded. The exceptions were Slovenia and Czechia, with respectively low and very low levels of poverty. The very low level of poverty in Czechia is a consequence of the government’s effective fight against unemployment and the activation of social transfers [47]. In Slovenia, the poverty level improved in 2019 compared to 2010. Ten years ago, the country was characterized by high levels of poverty. In the case of Poland, the poverty level slightly improved in 2019, mainly as a result of an improvement in the labor market and an increase in social transfers. In Slovakia, on the other hand, the poverty level has worsened compared to 2010. The situation was no better in the countries of southern Europe, such as Spain, Portugal, Cyprus, and Croatia, where the poverty level was also high. In most of these countries, the poverty reduction situation has not improved over the last decade. Malta was an exception. The poverty level in this country was very low. Apart from Malta, low poverty levels were characteristic of countries such as Austria and Czechia. Considering the 2010 ranking of countries in terms of the discussed values of the poverty level in two countries, such as Austria and Malta, an improvement in the situation is noticeable in 2019. The latest report of the Institute for European Environmental Policy [60] shows that in terms of achieving the goal of ending poverty in all its forms, Austria and Czechia have fully achieved this goal and are on their way to permanently maintain it.

The most homogeneous group of countries in terms of the level of poverty was the Nordic countries (Sweden, Finland, and Denmark). In these countries, poverty has remained relatively constant over the past decade. This situation should come as no surprise as the Nordic countries are said to be welfare states. In addition, the overriding goal of these states is to care for their citizens. In other words, these countries try to provide their inhabitants with such conditions that, even in a difficult life situation, they can afford to function at a suitable level. Moreover, these countries have the highest ranking of any country in the world in terms of The Human Development Index [61].

Our analysis also allows us to see certain divisions and shows uneven levels of poverty in the countries belonging to the old and the new Union. Of the analyzed 14 countries belonging to the old EU, in 2019, more than half was characterized by a low level of poverty, including one country, Austria, very low. In the case of 15 countries of the new EU, the poverty level in most of them was high or very high. This is related, inter alia, to the level of economic growth in these countries. Economic growth is the most powerful instrument in reducing poverty and improving the quality of life [62]. Sabir and Tahir [63] noted that economic growth means an increase in the ability of the economy to meet the needs and desires of society. It can generate positive circles of prosperity and opportunities and thus foster human development, which in turn fosters economic growth. Analyzing the dynamics of extreme poverty (USD $1.90 per day on the poverty line), Bergstrom [64] states that 90% of the volatility in poverty rates can be explained by changes in GDP per capita. As for the economies of the old EU countries, they are much more developed, which translates into a higher value of GDP, and thus contributes to achieving lower poverty levels in these countries.

Our discoveries contribute to contemporary debates on the effectiveness of implementing the most important goal of sustainable development, which is the eradication of poverty in all its forms. This study proves that most EU countries are not sustainable, and without taking urgent action in the area of poverty eradication, sustainable development in social, economic, and environmental terms seems to be impossible to achieve in the near future. COVID-19 pandemicis undoubtedly a challenge for taking effective measures to reduce the level of poverty. As David Nabarro, Special Representative of the UN Secretary General on COVID, “Like it or not, COVID is a disease of poverty, powerlessness, inequality and injustice—a disease of the disadvantaged—and is taking root in the poorest communities.” Forecasts indicate that the crisis caused by the pandemic may increase the number of people at risk of poverty, especially in the countries of the new EU, and the most vulnerable to its consequences will be the inhabitants of southern Europe [47], i.e., those countries where the poverty level in 2010 and 2019 was at a high or very high level.

## 5. Conclusions

The study assesses the scale of poverty in EU countries in 2010 and 2019. The goal was achieved on the basis of data obtained from the Eurostat database, which refers to the indicators characterizing SDG1—“No poverty”. Modified data were also used (winsorized data), which was obtained as a result of improving the quality of outliers. This procedure is justified when the distributions of the variables are asymmetric, and there are outliers. The use of winsorized data increased the range of the value of the synthetic measure, which allowed for the correct identification of objects in terms of the adopted criterion, i.e., the poverty scale.

The conducted research shows that Bulgaria, Romania, and Estonia are characterized by variables whose values differ significantly from the others, stimulating a high level of poverty. There are also countries, such as Malta or Luxembourg, for which the values of the variables are considered distant but of a low poverty level. The group of countries with the highest poverty level includes: Bulgaria, Romania, Lithuania, and Latvia, both in 2010 and 2019. The high level of poverty was recorded, among others, in countries such as: Croatia, Cyprus, Estonia, Poland, Portugal, and Spain. In the following countries: Austria, Czechia, Germany, Ireland, Luxembourg, Malta, Netherlands, Slovenia, Sweden, poverty was low or medium. This group includes mainly countries that have been EU members from the beginning of its existence. By contrast, the countries with high poverty levels are mainly those that joined the EU last.

Analyzing the spatial differentiation of countries in terms of the scale of poverty, it can be stated that half of the countries surveyed with low or very low poverty are countries located mainly in western Europe and Scandinavia. On the other hand, countries included in the high or very high poverty group are located in eastern and southern Europe. The obtained division of EU countries into typological classes indicates the scale of poverty is reflected in the value of GDP per capita in PPS as a percentage EU average. In the group of countries with a very high level of poverty, the lowest level of GDP per capita in PPS as a percentage EU average was recorded, while significantly higher values of GDP per capita in PPS as a percentage EU average were recorded in countries with a low or very low level of poverty.

Measuring poverty according to the severity of the problem should be made constantly. Future research on the measurement and assessment of poverty levels in European countries should repeat the existing ones in order to identify further progress or delays in its implementation in individual EU countries. This is important due to the large volatility of social, economic, and environmental conditions that all countries are currently facing, not only in Europe but also in the world. The impact of COVID-19 has three main factors. First, the direct impact of the disease itself in terms of the number of people infected with the virus, deaths, and excess mortality. Second, a direct impact on welfare, health, socio-economic, environmental, and civil rights due to measures taken by governments to slow down and stop disease, mainly lockdown. Third, the longer-term environmental, social, and economic impacts of COVID-19, global lockdown, and remedial measures that have not yet been fully experienced by society [65]. It would therefore be useful to analyze how the COVID-19 pandemic affected poverty levels in European countries. In particular, whether it was an impulse to intensify activities in the field of poverty eradication, or, on the contrary, it inhibited their implementation. There is also a need for more in-depth research to establish the mechanisms, direction, and causality underlying the achievement of the SDG1 goal and its interaction with other SDGs. Filling the gap in this area will additionally provide the basis for future evidence-based sustainable development policy.

## Figures and Tables

**Table 1:** No poverty indicators.

No.	Indicator (%)	Definition of the Indicator
1.	People at risk of poverty or social exclusion	The indicator corresponds to the sum of persons who are: at risk of poverty after social transfers, severely materially deprived, or living in households with very low work intensity. Persons are counted only once, even if they are affected by more than one of these phenomena. This is the multidimensional poverty index. The next three indicators are part of the multidimensional poverty index.
2.	People at risk of income poverty after social transfers	People at risk of poverty are persons with an equivalized disposable income below the risk-of-poverty threshold, which is set at 60% of the national median equivalized disposable income (after social transfers).
3.	Severely materially deprived people	The indicator measures the share of severely materially deprived persons who have living conditions severely constrained by a lack of resources. They experience at least 4 out of 9 following deprivations items: cannot afford: (1) to pay rent or utility bills, (2) keep home adequately warm, (3) face unexpected expenses, (4) eat meat, fish or a protein equivalent every second day, (5) a week holiday away from home, (6) a car, (7) a washing machine, (8) a color TV, or (9) a telephone.
4.	People living in households with very low work intensity	The indicator is defined as the share of people aged 0–59 living in households with very low work intensity. These are households where on average, the adults (aged 18–59, excluding students) worked 20% or less of their total work potential during the past year.
5.	In work at-risk-of-poverty rate	The indicator measures the share of persons who are employed and have an equivalized disposable income below the risk-of-poverty threshold, which is set at 60% of the national median equivalized disposable income (after social transfers). For the purpose of this indicator, an individual is considered as being employed if he/she was employed for more than half of the reference year. The indicator is based on the EU-SILC (statistics on income, social inclusion, and living conditions).
6.	Population living in a dwelling with a leaking roof, damp walls, floors, or foundation or rot in window frames of floor by poverty status	The indicator measures the share of the population experiencing at least one of the following basic deficits in their housing condition: a leaking roof, damp walls, floors or foundation, or rot in window frames or floor.
7.	Self-reported unmet need for medical examination and care	The indicator measures the share of the population aged 16 and over reporting unmet needs for medical care due to one of the following reasons: (1) financial reasons, (2) waiting list, and (3) too far to travel. Self-reported unmet needs concern a person’s own assessment of whether he or she needed medical examination or treatment (dental care excluded) but did not have it or did not seek it. The data stem from the EU Statistics on Income and Living Conditions (EU-SILC).
8.	Population having neither a bath, nor a shower, nor indoor flushing toilet in their household by poverty status	The indicator measures the share of total population having neither a bath, nor a shower, nor an indoor flushing toilet in their household.
9.	Population unable to keep home adequately warm by poverty status	The indicator measures the share of population who are unable to keep home adequately warm. Data for this indicator are being collected as part of the European Union Statistics on Income and Living Conditions (EU-SILC) to monitor the development of poverty and social inclusion in the EU.
10.	Overcrowding rate by poverty status	The indicator measures the share of people living in overcrowded conditions in the EU. A person is considered to be living in an overcrowded household if the house does not have at least one room for the entire household as well as a room for a couple, for each single person above 18, for a pair of teenagers (12 to 17 years of age) of the same sex, for each teenager of different sex and for a pair of children (under 12 years of age).

**Table 2:** Values of basic descriptive parameters of no poverty indicators for EU countries in 2010 and 2019 for winsorized data.

Year		Mean	Median	Min	Max	St. Dev.	Skewness	Kurtosis
1.	2010	23.83	21.70	14.40	34.90	6.40	0.45	−0.96
	2019	21.09	20.10	12.50	31.60	5.16	0.57	−0.46
2.	2010	15.99	15.50	9.00	21.60	3.53	0.05	−0.91
	2019	16.33	15.40	10.10	23.80	3.94	0.39	−1.00
3.	2010	9.40	6.50	0.50	21.50	6.72	0.80	−0.59
	2019	5.54	4.70	1.30	11.90	3.31	0.67	−0.72
4.	2010	9.24	9.00	5.40	13.10	2.22	0.23	−0.73
	2019	7.85	7.60	4.20	11.80	2.28	0.21	−0.91
5.	2010	7.69	7.20	3.70	12.80	2.76	0.55	−0.76
	2019	7.78	7.80	2.90	13.70	2.93	0.27	−0.71
6.	2010	15.76	17.10	6.50	20.50	4.67	−0.73	−0.64
	2019	12.91	12.50	4.10	19.90	4.54	0.00	−0.69
7.	2010	3.14	1.90	0.10	8.30	2.76	0.88	−0.55
	2019	1.94	1.40	0.00	4.70	1.60	0.73	−0.78
8.	2010	1.77	0.40	0.00	6.60	2.56	1.28	−0.14
	2019	0.86	0.30	0.00	2.70	1.07	1.06	−0.64
9.	2010	10.03	6.80	0.50	24.10	7.98	0.73	−0.88
	2019	6.61	5.40	1.80	14.40	4.53	0.74	−0.82
10.	2010	23.16	14.60	2.00	55.70	18.69	0.41	−1.53
	2019	17.98	13.90	2.20	44.90	13.80	0.74	−0.90

**Table 3:** Values of the synthetic measure and the rank of EU countries in terms of level of poverty in 2010 and 2019.

Countries	Values of Syntethic Measures		Rank of Countries		Level of Poverty	
	2010	2019	2010	2019	2010	2019
Austria	0.298	0.259	22	25	medium	low
Belgium	0.385	0.403	14	15	medium	medium
Bulgaria	0.724	0.696	4	3	very high	very high
Croatia	0.593	0.474	6	13	high	high
Cyprus	0.471	0.475	12	12	high	high
Czechia	0.206	0.136	27	27	low	low
Denmark	0.256	0.339	24	19	low	medium
Estonia	0.542	0.522	9	7	high	high
Finland	0.235	0.333	25	20	low	medium
France	0.303	0.321	21	21	medium	medium
Germany	0.352	0.279	17	24	medium	low
Greece	0.566	0.655	8	4	high	very high
Hungary	0.569	0.492	7	9	high	high
Ireland	0.371	0.377	15	16	medium	medium
Italy	0.537	0.590	10	6	high	high
Latvia	0.864	0.707	1	1	very high	very high
Lithuania	0.745	0.620	3	5	very high	very high
Luxembourg	0.323	0.367	19	17	medium	medium
Malta	0.334	0.256	18	26	medium	low
Netherlands	0.229	0.295	26	23	low	medium
Poland	0.651	0.477	5	11	very high	high
Portugal	0.533	0.511	11	8	high	high
Romania	0.787	0.701	2	2	very high	very high
Slovakia	0.310	0.412	20	14	medium	medium
Slovenia	0.362	0.342	16	18	medium	medium
Spain	0.466	0.479	13	10	high	high
Sweden	0.256	0.307	23	22	low	medium
Max	0.864	0.707				
Min	0.206	0.136				
Range	0.658	0.571				
Average	0.454	0.438				
Coefficient of variation (%)	41.192	34.872				

**Table 4:** Typological classification of EU countries in terms of poverty level in 2010 and 2019.

Group	Level of Poverty	Approaches			
		2010		2019	
		Number of Class	%	Number of Class	%
I	Very high	5	18.5	5	18.5
II	High	8	29.6	8	29.6
III	Medium	9	33.3	10	37.0
IV	Low	5	18.5	4	14.8

## Data Availability

Data were obtained from https://ec.europa.eu/eurostat (accessed on 11 December 2021).
